# Pre‐Sealing of Endodontic Access Cavities for the Preservation of Anterior Teeth Fracture Resistance

**DOI:** 10.1002/cre2.936

**Published:** 2024-07-17

**Authors:** Fereshteh Shafiei, Maryam S. Tavangar

**Affiliations:** ^1^ Department of Operative Dentistry, School of Dentistry, Oral and Dental Disease Research Center Shiraz University of Medical Sciences Shiraz Iran

**Keywords:** composite resins, ethylenediaminetetraacetic acid, root canal preparations, sodium hypochlorite solution

## Abstract

**Objective:**

Sodium hypochlorite solution (NaOCl) is an effective canal irrigant but interferes with the mechanical features of dentin and the bonding capability of adhesives when restoring endodontically treated teeth. This study evaluated whether access cavity resin sealing before using canal irrigant would augment the resistance of endodontically treated anterior teeth against fracture.

**Methods:**

Sixty maxillary incisors underwent endodontic treatment in five groups (*n* = 12). Irrigation with 5.25% NaOCl and 17% ethylenediaminetetraacetic acid (EDTA) was performed in all groups except for Group 5. After root canal obturation, in Group 1, the access cavity was kept unrestored. In Group 2, immediate restoration after obturation was achieved. For Group 3, delayed restoration after 1 week was provided. In Group 4 (pre‐sealed), before canal irrigation, the dentin surface of access cavities was sealed using self‐adhesive composite resin (Vertise Flow) and then restored after obturation. In Group 5, which was saline irrigated, immediate restoration was performed. After storage and thermal cycling for 5000 cycles at 5°C–55°C with a dwell time of 15 s and a transfer time of 5 s, teeth were statically loaded by a universal testing machine until a fracture occurred. Data were collected as the fracture resistance (FR) and analyzed using the one‐way analysis of variance and Tukey's tests.

**Results:**

FR significantly differed between all groups (*p* < 0.001). The lowest FR was recorded in the unrestored group (284 ± 86 N), which was not statistically different from the immediately restored group (*p* = 0.065). The pre‐sealed group exhibited the highest FR value (810 ± 127 N, *p* ≤ 0.02 vs. other groups). The FR of the saline‐irrigated and delayed restored groups was almost similar (*p* = 0.13).

**Conclusions:**

NaOCl/EDTA irrigation resulted in an adverse effect on FR. Delayed restoration could reduce this adverse effect. Access cavity pre‐sealing with flowable composites led to a higher FR than conventional methods and may be considered an effective step during treatment procedures.

## 1 Introduction

Restorative materials can seal endodontically treated teeth to avert premature coronal leakage and achieve a successful endodontic treatment (De Rose, Krejci, and Bortolotto 2015; Ebert et al. 2009; Uranga et al. 1999). The dentin of endodontically treated teeth undergoes some structural alterations, such as water loss and collagen cross‐linking weakening (Comba et al. 2021). Consequently, the weakened biomechanical behavior of tooth structure of endodontically treated teeth makes their restoration challenging for dental clinicians (Comba et al. 2021). Compared to nonadhesives, adhesive restoration, such as composite resin, presents greater fracture resistance (FR) by reinforcing the remaining tooth structures (Soares et al. 2008). Lacking an adhesive bond, nonadhesive restorations achieve less FR and more treatment failures (Soares et al. 2008). In addition, when nonadhesive restorations such as amalgam were used in combination with adhesive resins, the FR of teeth after endodontic treatment would reach similar values to those of composite resin restorations (Eakle, Staninec, and Lacy 1992; Sagsen and Aslan 2006). These findings may demonstrate the role of adhesion in increasing the FR of teeth after endodontic treatment.

The bonding capacity in adhesive restoration is affected by substrate conditioning or pretreatments. Some studies have reported that sodium hypochlorite solution (NaOCl), which is applied commonly as the most effective irrigant, might interfere with the dentin bonding capability of adhesive systems (Fawzi, Elkassas, and Ghoneim 2010; Gönülol, Kalyoncuoğlu, and Ertaş 2015; Ozturk and Ozer 2004; Santos et al. 2006). The association of ethylenediaminetetraacetic acid (EDTA) did not minimize this adverse effect (Santos et al. 2006). EDTA can remove calcium ions from root dentin (de Andrade Marafiga et al. 2022) causing erosion of intertubular and peritubular dentin. Excessive mineral loss may occur due to the strong chelation ability of EDTA (Ulusoy, Mantı, and Çelik 2020). Hence, it adversely affected the mechanical properties of root dentin, such as microhardness, and flexural and cohesive resistance (Antunes et al. 2020; de Andrade Marafiga et al. 2022). Moreover, collagen degradation was demonstrated in EDTA–treated dentin (Pheenithicharoenkul and Panichuttra 2016). In addition, during irrigation procedures, long exposure time to high concentrations of NaOCl with proteolytic properties could decrease calcium and phosphate levels, microhardness, and elastic modulus and flexural strength of dentin structure (Marending et al. 2007; Moreira et al. 2009; Durigon et al. 2020; Gu et al. 2017). In a study, it was shown that NaOCl irrigant may cause a reduction in the FR of the tooth. This finding may be explained by the mineral loss in dentin as a consequence of the OCl anion‐induced acidification. This newly damaged dentin poses a brittle matrix that is less receptive to adhesive resins, resulting in a poor micromechanical interaction between dentin and adhesives (Santos et al. 2006). Moreover, NaOCl can inhibit resin polymerization (Moreira et al. 2009).

Nevertheless, due to its unique bactericidal and debriding effects (Mohammadi 2008), NaOCl is difficult to replace with an alternative irrigant. In addition to the root canal dentin, the access cavity's dentinal surfaces are also exposed to the NaOCl irrigant solutions. A possible approach to reduce the aforementioned shortcomings could be the protection of freshly cut dentin from the irrigants and the establishment of adhesive resin bonding in the access cavity before irrigation (De Rose, Krejci, and Bortolotto 2015). This idea in endodontic treatment originated from immediate dentin sealing (IDS), which is well‐documented (Magne, So, and Cascione 2007). In a previous study, it was revealed that IDS of the endodontically treated tooth prepared for composite resin inlay strengthens the teeth restored with adhesive technique (Shafiei, Aghaei, and Jowkar 2020). Immediate endodontic sealing or pre‐sealing of the access cavity (De Rose, Krejci, and Bortolotto 2015) versus conventional or delayed endodontic sealing (Ebert et al. 2009) was found to improve the “internal adaptation of composite resin restoration” in the mesio‐occlusal cavity of the molars (De Rose, Krejci, and Bortolotto 2015).

In light of the aforementioned studies, it can be hypothesized that pre‐sealing of the freshly cut dentin in the access cavity would possibly result in an increased FR of the composite resin‐restored teeth. As this hypothesis has not previously been assessed, we aimed to examine the effect of NaOCl/EDTA irrigation and pre‐sealing the access cavity on the FR of resin‐restored central incisors. In the present study, a self‐adhesive flowable composite resin will be used to create a pre‐sealed dentin layer. This type of composite is characterized by low viscosity, as its flowability facilitates penetration into the dentin structures achieving an acceptable seal and adaptation (Yao et al. 2020). The elimination of bonding systems can provide an easy application, simplified usage steps, lower technical sensitivity, and minimized time for possible contamination compared to conventional bonding agents and resin composites (Alshabib et al. 2023; Memarpour et al. 2016), saving time and reducing bond application mistakes (Yuan et al. 2015).

## 2 Materials and Methods

We commenced the study following our university ethics committee's approval (Code: IR.SUMS.DENTAL.REC.1398.023). Sixty intact human periodontally involved mature maxillary central incisors were used. After cleaning the teeth and disinfecting them with 0.5% chloramine solution, we stored them at 4°C in distilled water for use within 2 months. Similar teeth were selected, varying by a maximum of 0.5 mm in each target dimension (mesiodistal: 8.4 mm; buccopalatal: 7.3 mm; length: 9.2 mm) and lacking cracks/fractures or caries.

We calculated the sample size based on a previous study (Fadag 2018) considering the amount of fracture load, an *α* value of 0.05, and 80% study power. After corrections, 60 samples were required, with 12 teeth randomly allocated to each of the following groups:

Group 1 (unrestored): The access cavity was kept unrestored after root canal treatment and served as the negative control.

Group 2 (immediately restored): Immediate restoration was performed after finishing the root canal treatment.

Group 3 (delayed restored): Delayed restoration was performed 1 week after finishing the root canal treatment.

Group 4 (pre‐sealed): Before using irrigants, we sealed the access cavity's dentin surfaces using self‐adhesive composite resins. Then, we applied immediate restoration after root canal treatment.

Group 5 (saline irrigated): Irrigated with normal saline instead of NaOCl/EDTA restoration, and immediate restoration was applied after root canal treatment (positive control).

### 2.1 Root Canal Treatment

To prepare the traditional access cavity, we removed the pulp chamber roof, pulp horns, and lingual shoulder of dentin and standardized it with a #4 steel round bur. The pulpal chamber was completely cleaned from pulp tissue debris using an ultrasonic scaler. The teeth underwent endodontic treatment as follows: We used a ProTaper Sx rotary device (Dentsply Miallefer, Ballaigues, Switzerland) to enlarge the orifice and the canal's coronal third, rendering the middle and apical thirds accessible. The irrigation used in this initial step was only normal saline. Using a #10 K‐file, we established that the working length was 1 mm short of the apical foramen. We prepared the roots with the rotary device up to #F3 in association with irrigation solutions. The standardized irrigation method was performed for teeth using a 5‐mL disposable plastic syringe and a 31‐gauge side‐vented needle, with the needle placed into the canal without binding (Reddy et al. 2018). The quantity and contact time of each solution were standardized to 5 mL of 5.25% NaOCl (ChloraXiD, PPH Cerkamed, Stalowa‐Wola, Poland) for 1 min after each file, then 5 mL of 17% EDTA (Pulpdent, Watertown, MA, USA) for 2 min, and finally 5 mL of 5.25% NaOCl for 1 min to remove the smear layer (Özyürek et al. 2018). This irrigation protocol was followed for all groups except Group 5 (saline‐rinsed; positive control), in which the only irrigant used was normal saline with the same contact time as the other groups.

Afterward, across all groups, we dried the canals using paper points and filled them using gutta‐percha (ProTaper, Dentsply Sirona, Dentsply Maillefer, Ballaigues, Switzerland) and a resin‐based sealer (AH Plus, Dentsply De Trey, Konstanz, Germany) via the “single‐cone technique.” We removed the coronal root fillings until 1 mm beneath the cementoenamel junction (CEJ) with the aid of a heated plugger.

### 2.2 Method of Pre‐Sealing

In Group 4 (pre‐seal), before irrigation (as mentioned above), the access cavity's dentin surfaces were cleaned with an ultrasonic scaler and a foam pellet moistened with ethanol and disinfected with 2% chlorhexidine solution, rinsed using water, and then air‐dried. Subsequently, we applied a 0.5‐mm‐thick layer of self‐adhesive flowable composite resin (Vertise Flow, Kerr, USA) to the access cavity's dentin surface as instructed by the manufacturer and light‐cured for 40 s. Before this, the canal orifice was blocked and protected from resin sealing by the insertion of a compacted cotton pellet. Instrumentation and irrigation were then carried out with the same irrigation protocol as the other groups.

### 2.3 Methods of Restoration

In Group 1, the access cavity was kept unrestored and served as the negative control. In Groups 2–5, access cavities were restored. The access cavity was cleaned with a foam pellet moistened with ethanol. The cavity was sealed and restored with etch‐and‐rinse adhesive (Adper Single Bond) along with Filtek Z250 composite resin (3M ESPE), as the conventional clinical method. Using an incremental approach, we performed composite resin restoration. Each increment was light‐cured for 40 s with a light‐curing machine (VIP Junior, Bisco, Schaumburg, IL, USA) set at 600 mW/cm^2^ intensity. All the restorations were finished and polished. Table 1 specifies the applied restorative materials.

**Table 1:** Restorative materials used in this study.

Material or instrument	Type	Manufacturer
NaOCl (5.25%)	Irrigant	ChloraXiD, PPH Cerkamed, Stalowa‐Wola, Poland
EDTA (17%)	Irrigant	Pulpdent, USA
Gutta‐percha	Root canal filler	Dentsply Sirona, Germany
AH Plus	Resin‐based sealer	Dentsply De Trey, Germany
Vertise Flow	Self‐adhesive flowable composite resin	Kerr, Orange, CA
Adper Single Bond	Etch‐and‐rinse adhesive	3M ESPE, St. Paul, USA
Filtek Z250	Composite resin	3M ESPE, St. Paul, USA
#4 steel round bur	Bur	Jota, Germany
Mini Piezon	Ultrasonic scaler	EMS, Switzerland
ProTaper Sx	Rotary instrument system	Dentsply Miallefer, Ballaigues, Switzerland
#10 K‐file	Endodontic file	Dentsply‐Maillefer
VIP Junior	Light‐curing unit	Bisco, Schaumburg, IL, USA

### 2.4 FR Measurement

After 6 months of storing the teeth in water at 37°C with thermal cycling for 5000 cycles at temperatures ranging from 5°C to 55°C with a dwell time of 15 s in each bath and a transfer time of 5 s, they were mounted in cylinder‐shaped molds of self‐cured acrylic resin up to 2 mm apical to the CEJ. Before mounting, we used melted wax (a layer measuring 0.2–0.3 mm) to cover the roots. We mimicked the periodontal ligament by replacing this wax layer with silicone impression material.

**Figure 1:**
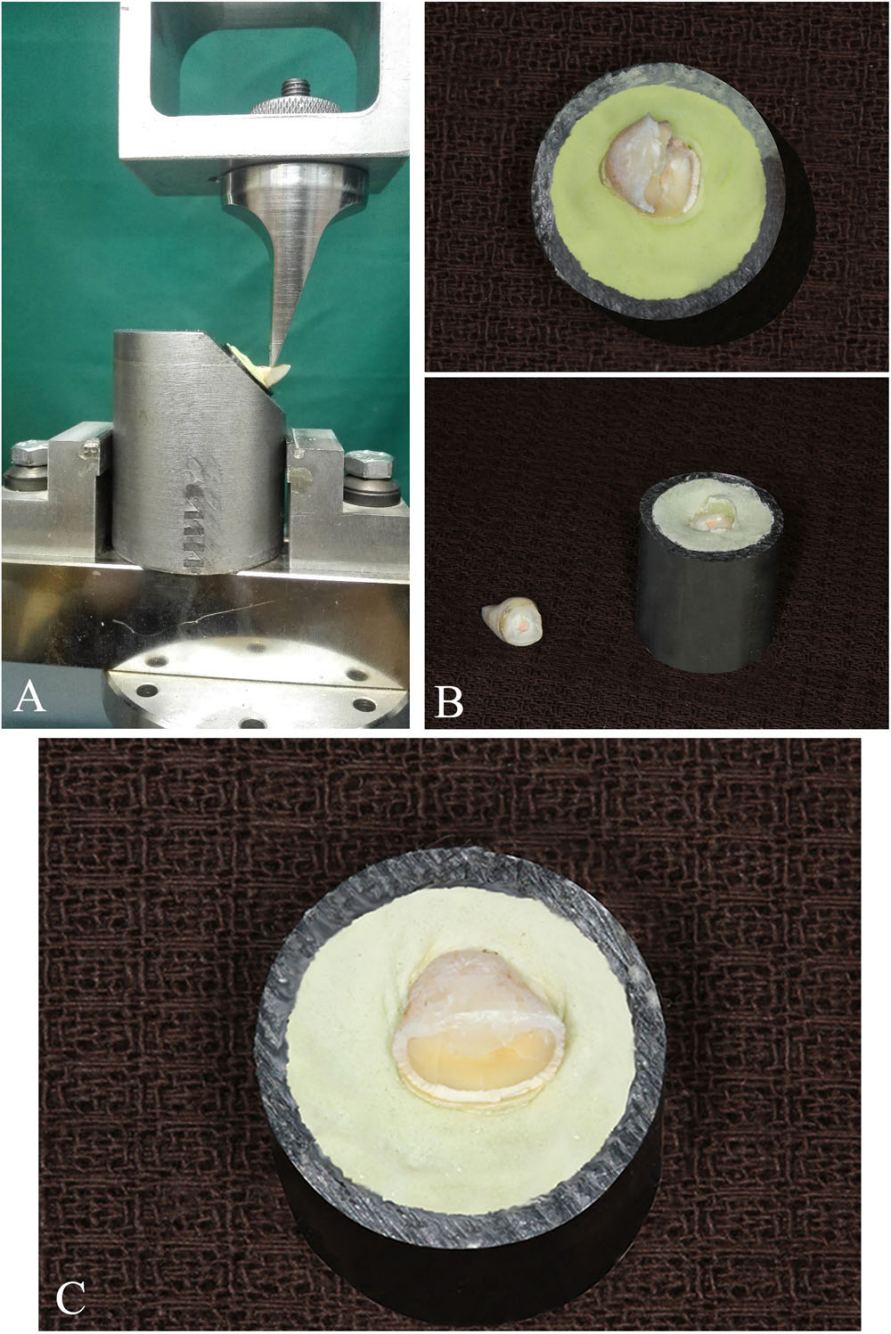
(A) Fracture resistance test using a chisel‐shaped blade on the lingual surface at a 130° angle with the long axis of the root. (B) Unfavorable mode of fracture. (C) Favorable mode of fracture.

To measure FR, we used a universal testing machine (Zwick Roell, Ulm, Germany). We applied axial force above the cingulum at 135° relative to the root's long axis (Figure 1A) with 1 mm/min crosshead speed (Oskoee et al. 2018). Therefore, the teeth's static loading capacity until fracture occurrence was determined in Newtons (N).

### 2.5 Fracture Mode Evaluation

Two independent observers (M.S.T. and F.S.) evaluated the teeth following a fracture using a × 4 magnifying glass to classify the fracture mode as “favorable” if the fracture ended superior to the embedded resin or “unfavorable” if it ended beneath the embedded resin.

### 2.6 Statistical Analysis

We confirmed the normality of continuous data using the Kolmogorov–Smirnov test and then ran the one‐way ANOVA test, followed by Tukey's tests for pair‐wise comparisons. We used the *χ* ^2^ test to compare numerical data across groups. We considered significance at *p* < 0.05.

## 3 Results

### 3.1 FR

**Figure 2:**
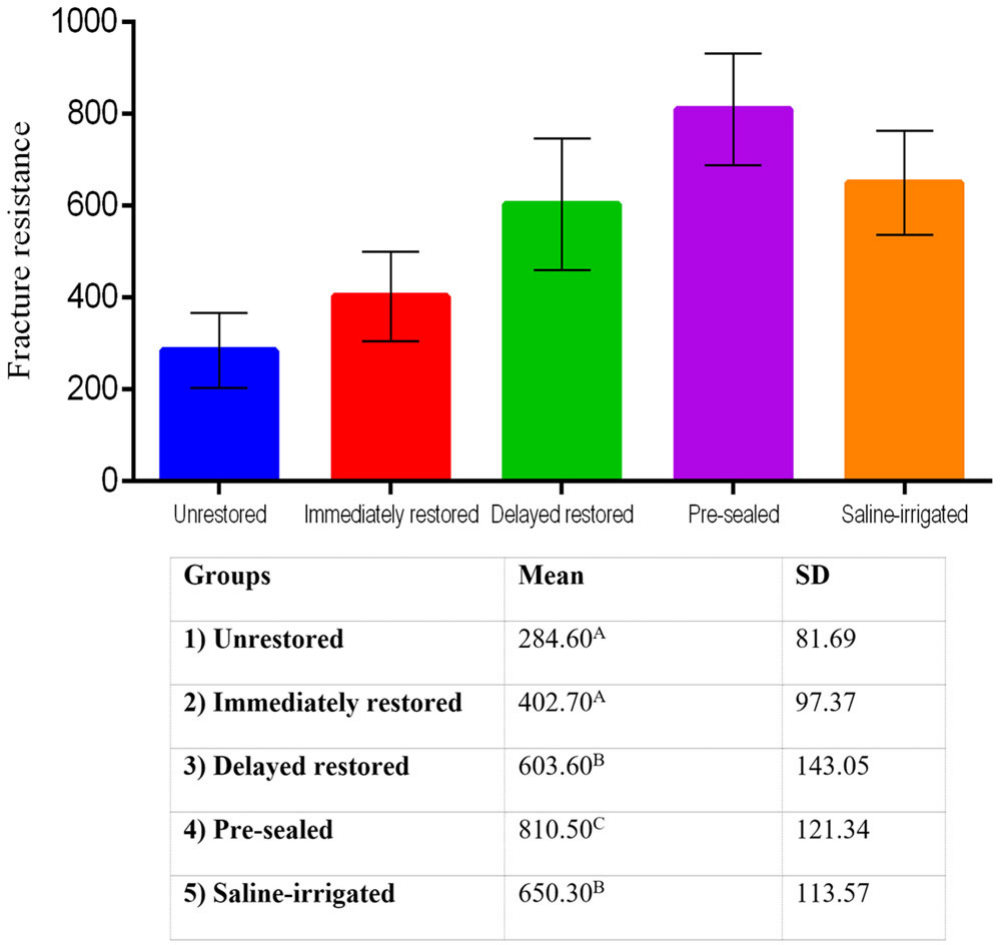
Fracture resistance (in Newton) of the study groups (*N* = 12). Different superscript capital letters indicate significant differences (*p* < 0.05) according to post hoc Tukey's test between groups.

Figure 2 summarizes the FR values, which differed significantly across the groups (*p* < 0.001). The lowest FR was recorded in the unrestored group (284 ± 86 N). This value was not statistically different from the values from the immediately restored group (402 ± 102 N; *p* = 0.065); both of the mentioned groups yielded significant differences relative to the other three groups (*p* ≤ 0.001 for all comparisons). The pre‐sealed group exhibited the highest FR value (810 ± 127 N), which showed a significant difference compared to all other groups (*p* ≤ 0.02). The FR of the saline‐irrigated and delayed restored groups was almost similar (*p* = 0.13).

### 3.2 Fracture Mode Analysis

The fracture mode across the five groups is summarized in Table 2. All samples of the unrestored group were fractured in the unfavorable mode (Figure 1B), while the favorable mode was predominant in the saline‐irrigated and pre‐sealed groups (Figure 1C). Both fracture modes were observed in the other groups.

**Table 2:** Mode of failure in the studied groups.

	Favorable	Unfavorable
Unrestored	0	12
Immediately restored	5	7
Delayed restored	6	6
Pre‐sealed	10	2
Saline‐irrigated	9	3

## 4 Discussion

According to our findings, FR was greater in the saline‐rinsed group than in the immediately restored group, rejecting the null hypothesis that NaOCl/EDTA does not affect the FR of restored incisors. Furthermore, self‐adhesive resin pre‐sealing of the access cavity considerably improved FR, rejecting the null hypothesis that pre‐sealing does not affect FR.

The FR of endodontically treated teeth depends on the amount of the remaining tooth structure, the adhesive surface, and the adhesion quality (Dietschi et al. 2007). As anterior restorations face “high masticatory loads and parafunctional forces,” fractures may occur over time. As a solution, adhesive measures at both radicular and coronal levels during endodontic treatment can reinforce the tooth and optimize its stability (Dietschi et al. 2007). In the radicular portion, the use of an adhesive sealer was recommended because it was thought that the adhesion of sealers to interradicular dentin and providing micromechanical interlocking might reinforce the structure and enhance the FR of endodontically treated teeth (Mohammed and Al‐Zaka 2020). Similarly, providing successful adhesion in the coronal structure can augment the FR of endodontically restored teeth (Spitznagel et al. 2014).

The reduced binding ability of adhesives to NaOCl–treated dentin may interfere with providing adequate restoration durability (Fawzi, Elkassas, and Ghoneim 2010; Marending et al. 2007; Moreira et al. 2009). Accordingly, antioxidants have been introduced to compensate for the oxidative effect of NaOCl and allow an immediate adhesive application with sufficient bond strength (Cecchin, Farina, and Bedran‐Russo 2018; Corrêa et al. 2016; Dikmen et al. 2015; Gönülol, Kalyoncuoğlu, and Ertaş 2015). However, the detrimental effect of NaOCl on the collagen matrix is still a problem. The application time of most antioxidants for the complete reversal of bond strength of adhesives was 5 or 10 min, which is too long in clinical practice. In addition, the application of antioxidants could cause tooth discoloration (Frassetto et al. 2016).

Some authors suggested postponing adhesive application/restoration for 1 week after endodontic treatment to neutralize the adverse effects of oxidative agents (Dikmen et al. 2015). This delay in the restoration was supported by our work as the FR of the delayed restoration group reached the level of the saline group and was higher than that of the immediately restored teeth. Nevertheless, this delayed restoration is not recommended as immediate coronal sealing is crucial following endodontic treatment (Heling et al. 2002; Sadaf 2020; Safavi, Dowden, and Langeland 1987). Unlike this result, Saber and El‐Askary (2009) found that the dentin bond strength of the restorations did not improve after a delay of 1 week. Consequently, other practical approaches that may prevent the adverse effects of the irrigants are welcome.

In the present study, we have demonstrated that self‐adhesive resin pre‐sealing of the access cavity not only recovered the reduced FR of immediate restorations but also considerably improved FR relative to the saline group. Moreover, favorable fractures predominated in the pre‐sealed group, affecting the prognosis of the restored teeth. Some mechanisms can be suggested to explain these findings. A pre‐sealing layer of the flowable composite resin can prevent the contact of high concentrations of NaOCl irrigants with the dentin surface. Thus, this intervention may protect the freshly cut dentin from the adverse mechanical effect of exposure to NaOCl. In addition, pre‐sealing the access cavity's freshly cut dentin surfaces may preserve the adhesion by inhibiting the possible adverse impact of NaOCl on the dentin bond strength to the adhesive restoration. Another explanation for the observed enhancement of FR in the pre‐sealed group is the flowable composite resin's characteristics. while the stabilizing resin–dentin bond in the deep and narrow form of the cavity is a clinical challenge, easy application and good adaptation of the pre‐sealing layer of the flowable composite may help to reduce the polymerization stress effect in this high C‐factor situation (da Silva et al. 2007).

During IDS after tooth preparation for indirect restoration, using a flowable composite resin layer over the adhesive resin‐sealed preparation is recommended (Abu‐Nawareg et al. 2015; Magne 2014). In our work, we applied a self‐adhesive flowable composite resin for pre‐sealing as it offers the combined advantages of using flowable composite resin, simplifying the bonding steps, and reducing the clinical time. In the pre‐sealed group, after root canal obturation, acid‐etching and rinsing might have removed any residual oxygen from the resin surface. Due to the short time interval between pre‐sealing and adhesive composite resin restoration, the chemical bond would be reliably provided. This might be an advantage for the pre‐sealing technique compared to the IDS method, which has a longer interval between immediate sealing and resin bonding (Qanungo et al. 2016).

Although NaOCl and EDTA solutions are the main irrigants used, different concentrations, amounts, application durations, and sequences have been employed in endodontic literature (Reddy et al. 2018). These variations justify different outcomes of the irrigants on adhesive efficacy in bonding to different dentin substrates. The irrigation protocol used in this study was based on the reports demonstrating improved effectiveness in microbial killing within the exposed dentinal tubules and enhanced penetration of the sealer following this successive irrigation (Özyürek et al. 2018; Reddy et al. 2018).

One limitation of pre‐sealing the access cavity could be that it resulted in the prevention of the antibacterial effects of the irrigation solutions used during endodontic instrumentation. Therefore, before pre‐sealing was used in this study, the access cavity was cleaned with an ultrasonic scaler and then irrigated with chlorhexidine. A comparison of NaOCl and chlorhexidine in terms of antibacterial efficacy revealed a similar effect or higher efficacy of chlorhexidine or NaOCl was reported in clinical trials (Gonçalves et al. 2016). Furthermore, in the FR test used in this study, we imposed force upon the lingual surface with the blade set at a 135° angle relative to the root's long axis. This position could mimic the interincisal angle between the anterior teeth of the mandible and maxilla. However, the static loading applied was not capable of simulating functional and fatigue loading in intraoral conditions. Further long‐term studies are suggested to validate the impact of resin pre‐sealing on the dentin bond strength and marginal adaptation of adhesive resin restorations.

## 5 Conclusion

Our in vitro study indicates that NaOCl/EDTA irrigation during endodontic treatment adversely affects the FR of immediately restored teeth. Restoration with a 1‐week delay can mitigate this adverse effect. Immediate pre‐sealing of the access cavity's dentin surface using a self‐adhesive flowable composite may be considered an effective step during treatment procedures to increase the FR of the composite resin‐restored anterior teeth compared to conventional methods. However, future clinical investigations are needed to confirm this finding in daily clinical practice.

## Author Contributions

Both authors contributed equally to all aspects of the research.

## Ethics Statement

The protocol of the present research was approved by the university ethics committee with Ethical Approval Number IR.SUMS.DENTAL.REC.1398.023.

## Conflicts of Interest

The authors declare no conflicts of interest.

## Data Availability

The data that support the findings of this study are available on request from the corresponding author. The data are not publicly available due to privacy or ethical restrictions.
